# Physical, clinical, and psychosocial parameters of adolescents with different degrees of excess weight☆

**DOI:** 10.1016/j.rpped.2014.04.003

**Published:** 2014-12

**Authors:** Vanessa Drieli Seron Antonini, Danilo Fernandes da Silva, Josiane Aparecida Alves Bianchini, Carlos Andres Lopera, Amanda Caroline Teles Moreira, João Carlos Locateli, Nelson Nardo

**Affiliations:** Universidade Estadual de Maringá (UEM), Maringá, PR, Brazil

**Keywords:** Overweight, Obesity, Adolescent health, Motor activity, Quality of life

## Abstract

**OBJECTIVE:**

To compare body composition, hemodynamic parameters, health-related physical fitness, and health-related quality of life of adolescents with anthropometric diagnosis of overweight, obesity, and severe obesity.

**METHODS:**

220 adolescents with excess body weight were enrolled. They were beginners in a intervention program that included patients based on age, availability, presence of excess body weight, place of residence, and agreement to participate in the study

**.:**

This study collected anthropometric and hemodynamic variables, health-related physical fitness, and health-related quality of life of the adolescents. To compare the three groups according to nutritional status, parametric and non-parametric tests were applied. Significance level was set at *p<*0.05.

**RESULTS:**

There was no significant difference in resting heart rate, health-related physical fitness, relative body fat, absolute and relative lean mass, and health-related quality of life between overweight, obese, and severely obese adolescents (*p>*0.05). Body weight, body mass index, waist and hip circumference, and systolic blood pressure increased as degree of excess weightincreased (*p<*0.05). Dyastolic blood pressure of the severe obesity group was higher than the other groups (*p<*0.05). There was an association between the degree of excess weight and the prevalence of altered blood pressure (overweight: 12.1%; obesity: 28.1%; severe obesity: 45.5%; *p<*0.001). The results were similar when genders were analyzed separately.

**CONCLUSION:**

Results suggest that overweight adolescents presented similar results compared to obese and severely obese adolescents in most of the parameters analyzed.

## Introduction

Recent data from the Brazilian Institute of Geography and Statistics (Instituto Brasileiro de Geografia e Estatística - IBGE)^1^ indicate that 20% of the population between 10 and 19 years have excess weight (overweight or obesity). This disease may bring health complications such as increased risk of cardiovascular disease as early as school age,^2^ type 2 diabetes,^3^ and reduction in physical, emotional, and social well-being.^4^

Levels of health-related physical fitness (HRPF) are inversely associated with the degree of excess weight in children and adolescents.^5^^,^ 6 Aires *et al* 6 observed an inverse correlation between body mass index (BMI) and maximal aerobic capacity in boys and girls with overweight and obesity. Levels of cardiorespiratory fitness and strength are also lower in adolescents with excess weight, compared to their normal-weight peers; however, there is no difference between overweight individuals in comparison to the obese.^5^

Another parameter that suffers a negative impact from obesity is health-related quality of life (HRQoL). Studies have found that excess weight is associated with lower HRQoL in adolescents.^4^^,^ 7 Poeta *et al* 8 found that obese adolescents had worse HRQoL scores for the physical, social, emotional, psychosocial, and total domains when compared to adolescents with normal weight. 

Although studies comparing overweight adolescents with normal weight adolescents show results that demonstrate the need for special attention to the young obese population, there are few comparisons between adolescents with different degrees of excess weight. In this context, Ricco *et al* 9 compared adolescents diagnosed with overweight and those with obesity, and found that overweight adolescents had similar health risks to the obese for values ​​of fasting blood glucose, oral glucose tolerance test (OGTT), total cholesterol, LDL-cholesterol, HDL-cholesterol and triglycerides.

Recently, Cole and Lobstein10 proposed cutoff points based on BMI classification for a further degree of excess weight in children and adolescents, which is known as severe obesity, based on a BMI of 35 kg/m² for adults. Children and adolescents classified as having severe obesity are at increased risk for metabolic syndrome, insulin resistance, triglycerides, and interleukin-6 when compared to the obese.^11^

However, as far as it is known, there are still few studies on the differences in anthropometric variables, body composition, hemodynamics, HRPF, and HRQoL in adolescents classified as having overweight, obesity, and severe obesity, and it is necessary to understand which health-related parameters a higher level of excess weight can influence. To know the variables that are most affected as the degree of excess weight increases can contribute to intervention strategies in the pediatric population with excess weight, suggesting greater attention to these parameters, whose worsening is directly related to a greater degree of excess weight. However, determining the variables for which overweight adolescents have similar results to adolescents with obesity and/or severe obesity will demonstrate the need to revisit health care policies for adolescents, which currently focus on young obese individuals.^9^

Thus, the aim of this study was to compare body composition, hemodynamic parameters, HRPF, and HRQoL in adolescents with anthropometric diagnosis of overweight, obesity, and severe obesity, considering all these outcomes as primary, given their relevance in the assessment of young obese individuals. 

## Method

This was a descriptive cross-sectional study of 220 adolescents with excess weight (overweight, obesity, or severe obesity) enrolled in a Multidisciplinary Obesity Treatment Program (MOTP) between the years 2009 and 2012. This is an intervention program that includes the participation of professionals from the areas of physical education, nutrition, psychology, and pediatrics, aiming at promoting positive changes on eating habits and physical activity in adolescents with excess weight through cognitive behavioral therapy. This program is offered twice a year (once every semester) and has a 16-week course of six-hour duration weekly activities. 

The program inclusion criteria were: a) age between 10 and 18 years; b) availability to participate in the interventions at the stipulated time and days; c) being overweight, obese, or severely obese, according to the cutoff points for BMI, age, and gender, proposed by Cole *et al*,^10^ d) residing in Maringa or its metropolitan area and; e) agreement to participate and with the informed consent (IC) signed by the adolescents and their parents/guardians from the document approved by the local Ethics Committee (Opinion No. 463/2009).

Exclusion criteria were: a) previously diagnosed genetic or endocrine diseases, reported to the pediatrician; b) long-term alcohol consumption; c) use of glucocorticoids and psychotropic drugs that could affect appetite. The same criteria were used to define participation in the study, except those regarding the availability to participate in interventions at the established time and days. Thus, 59 participants from the intervention program that did not meet the aforementioned criteria were excluded: one adolescent with type I diabetes and normal weight, according to BMI; one participant with intellectual impairment; 40 participants aged >18 years, and 17 aged <10 years. None of the patients involved in the study reported previous participation in a regular exercise program or systematic intervention for weight loss. The only regular physical activity reported by the participants was that performed during school physical education classes. 

A meeting was scheduled with those interested in participating in the project to explain the objectives and the types of interventions to which they would be submitted. Those who initially agreed to participate in the study signed the informed consent for the program, containing the information of assessments related to the present study. All study assessments were performed in the afternoon between 2 PM and 4 PM in spaces used for the program implementation. All adolescents were assessed at baseline, before initiating the program activities.

The adolescents were assessed, which included measurements of body weight, height, waist circumference (WC), and hip circumference (HC). Body weight was measured on a scale capable of measuring up to 300 kg, with a 0.05kgresolution. Height was measured with a stadiometer capable of measuring up to 2.30m with a 0.1cm resolution. BMI was calculated by dividing the weight of the adolescents by their height squared. The WC and HC were measured using an inextensible measuring tape capable of measuring up to 2m and with a 0.1cm resolution. The waist-hip circumference ratio (WHR) was calculated. 

Body composition assessment was performed using the InBody 520 multifrequency, octapolar, electrical bioimpedance device. Adolescents were advised to follow the recommendations described by Heyward^12^ for this type of assessment: fasting for at least two hours including water, urinating about 30 minutes prior to the evaluation; abstaining from consumption of caffeinated beverages in the previous 48 hours; avoiding intense physical efforts in the previous 24 hours; and, finally, avoiding use of diuretics during the previous seven days. Measures of absolute and relative fat mass (AFM and RFM) and absolute and relative lean mass (ALM and RLM) were included in the analysis. 

Sexual maturation was assessed according to Tanner stages^13^ through self-examination. Adolescents identified in Stage 1 were considered prepubertal, in stages 2 and 3 as pubertal, and in stages 4 and 5 as post-pubertal.

Resting heart rate (RHR) and blood pressure (BP) were measured after a period of 5 to 10 minutes of rest, using an electronic sphygmomanometer (Microlife(r); Aargau - Switzerland) which also measures heart rate. The measurement was made on the right arm using an adequate cuff size for the adolescent. The measurements were obtained in the sitting position. The prevalence of altered BP values​​ was determined from specific criteria for the studied population.^14^

The HRPF parameters were: flexibility, strength/endurance of the abdominal muscles, handgrip strength, and cardiorespiratory fitness.

The sit-and-reach test with Wells' Bench was used to evaluate flexibility, in which adolescents had to sit with their legs straight out and try to reach the greatest distance while both hands, one over the other, reach forward.^15^ The strength/endurance of the abdominal muscles were evaluated through the trunk flexion test, in which adolescents should perform as many repetitions of abdominal sit-ups possible during a period of 60 seconds.^15^ Handgrip strength was assessed by a Takey model TK 120142 dynamometer, with the adolescent standing, with legs slightly laterally spaced, arms alongside the body, wrist and forearm pronated, and the measurement scale facing the evaluator.^16^

Cardiorespiratory fitness was measured using the 20-meter back-and-forth test, which was initiated at 8.5km/h with progressive increments of 0.5km/h every minute until the subject reached exhaustion. The adolescents were instructed during the 20-meter run by a beep and a physical education professional who participated in order to help them regarding familiarization with the procedure and with the running pace. Estimated measurements of relative maximal oxygen uptake (VO_2_max) were used in the analysis.^17^

Regarding quality of life, the generic questionnaire PedsQL^TM^ 4.0 was applied for the adolescents' self-assessment. The questionnaire has 23 items covering: 1) physical functioning (eight items), 2) emotional functioning (five items), 3) social functioning (five items), and 4) school functioning (five items). The questions ask how much of a problem each item was during the last month, and respondents use a five-level response scale (0=never a problem; 1=almost never a problem; 2=sometimes a problem; 3=often a problem; 4=almost always a problem). The items were scored inversely and linearly translated into a 0-100 scale (0=100, 1=75, 2=50, 3=25, 4=0); thus, the higher the score, the better the HRQoL. 

Scaled scores were obtained according to the proponents' standardization. To create a summary score of psychosocial health scores (15 items), the mean was computed as the sum of items answered on the scales of emotional, social, and school dimensions divided by the number of items. To create a summary score of overall quality of life, the 23 items were computed, which includes the four domains of the tool.^18^ This questionnaire was validated into Portuguese by Klatchoian *et al* for children and adolescents (aged between 2 and 18 years).^19^ It was applied in a classroom with a capacity of about 30 adolescents, with the assistance of at least two examiners. 

Sample size calculation was based on a test power of 80%, an alpha of 5%, and a difference between the BMI of overweight adolescents (25.10±2.62kg/m²) and adolescents with severe obesity (33.99±5.17kg/m²), according to the results observed by Rizzo *et al*.^20^ Based on this calculation, the sample size for each group should be of at least 31 adolescents.

The Shapiro-Wilk test was used to verify normality and Levene's test to verify homogeneity. Data are presented as mean and standard deviation, and for the analysis of comparison between the three groups, according to their nutritional status, the Kruskal-Wallis test was used for data that did not show normal distribution and/or homogeneity, and one-way ANOVA was used for data that showed normality and/or homogeneity. When there were differences in the Kruskal-Wallis test, the LSD multiple comparison test was applied, whereas the Bonferroni multiple comparison test was applied for differences observed by one-way ANOVA. The analyses were performed using SPSS statistical software, release 13.0. The level of significance was set at *p*<0.05. 

## Results

Of the 220 assessed adolescents, 58 (26.4%) were classified by BMI as overweight, 96 (43.6%) as obese, and 66 (30.0%) as severely obese. In the group of overweight adolescents, 34 (58.6%) adolescents were girls, while in the groups of obese and severely obese patients, 50 (52.1%) and 32 (48.5%) adolescents were females. The mean age of the adolescents with overweight, obesity, and severe obesity was 13.2±1.9 years, 13.1±1.9 years, and 13.3±1.8 years, respectively, with no significant differences (*p*=0.646). Sexual maturation was evaluated in a partial sample (n=127). In the group of overweight adolescents, 57.5% were post-pubertal and 42.5% were pubertal. In the group of obese adolescents, 50% were post-pubertal and 50% were pubertal. As for the severely obese adolescents, 60% were post-pubertal, 37.1% were pubertal, and 2.9% were prepubertal. There was no association between sexual maturation stage and degree of overweight among the adolescents (*p*=0.949). 

In the total sample, statistical differences were found between the groups for the variables body weight, BMI, WC, HC, and SBP, in which overweight adolescents had lower values ​​than the group of obese and severely obese individuals. For the variables WHR and DBP, no differences were observed between the groups with overweight and obesity, but only between overweight and severe obesity. No difference was observed for height and RHR. The results for males and females analyzed separately were similar (Table 1). Considering the observed difference in SBP and DBP between the degrees of overweight, the prevalence of high blood pressure was determined in the total sample and according to the gender of the adolescent. It was observed that 12.1%, 12.5%, and 11.8% of the adolescents from the total sample, boys and girls with overweight, also had altered BP. 

**Table 1:**
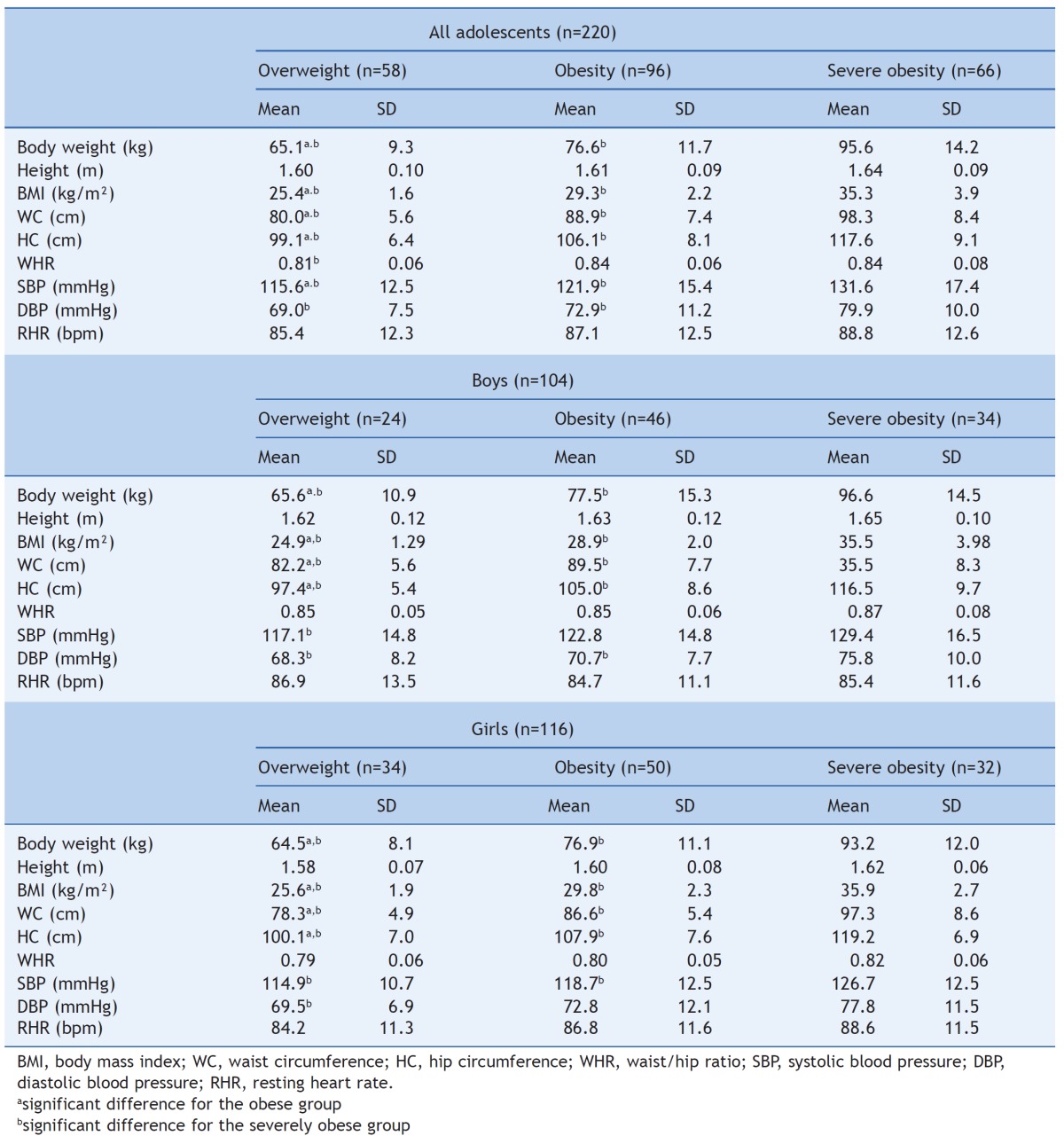
C Comparison of overweight, obese, and severely obese adolescents, regarding the anthropometric variables and hemodynamic parameters.

For the obese adolescents, the prevalence of alteration was 28.1%, 39.1%, and 18.0% in the entire sample, in boys, and in girls, respectively. For adolescents with severe obesity, the prevalence of alteration was 45.5%, 47.1%, and 43.8% in the total sample, in boys, and in girls, respectively. The degree of excess weight was associated with the prevalence of high blood pressure in the three conditions analyzed (total sample: *p*<0.001; boys: *p=*0.009; girls: *p*=0.002). 

Table 2 shows the values ​​for the variables of body composition and HRPF, and Table 3, the results found for the HRQoL domains. There was no significant difference when the three groups were compared, except for body fat (kg), in which the overweight group showed significantly lower results than adolescents classified as severely obese. When separated by gender, no difference was observed for any of the parameters of Tables 2 and 3. 

**Table 2:**
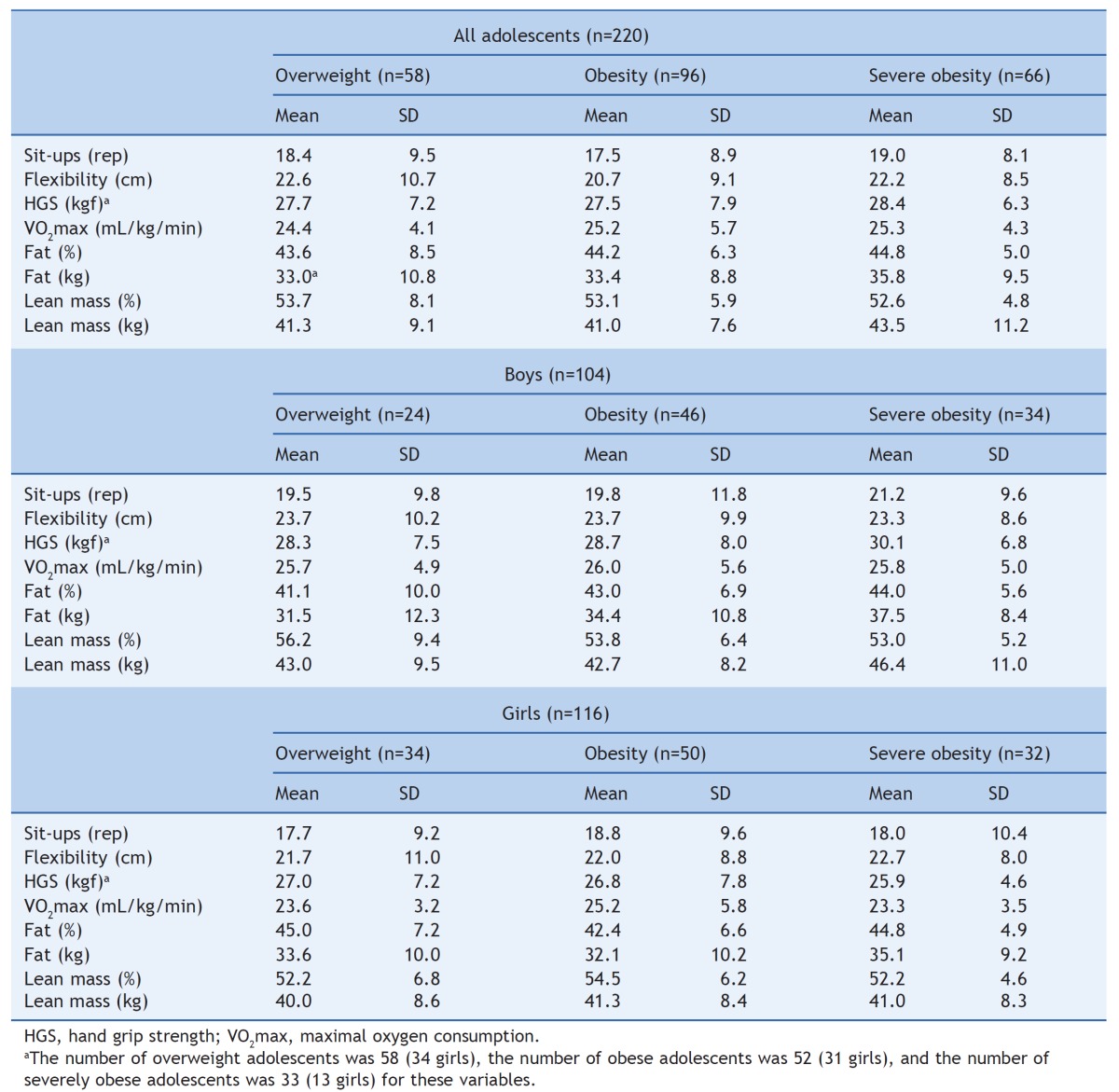
C Comparison of overweight, obese, and severely obese adolescents regarding the variables of health-related physical fitness and body composition.

**Table 3:**
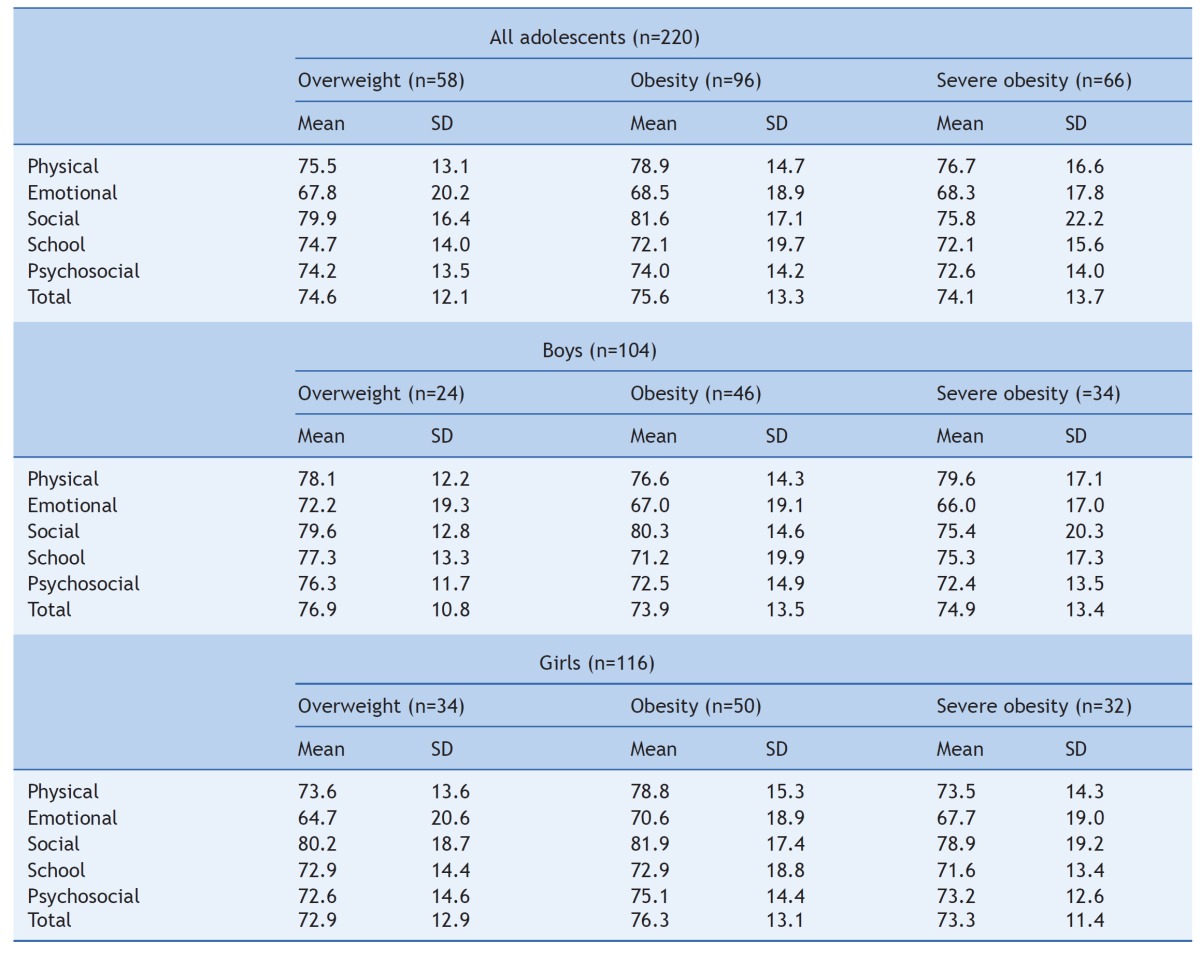
C Comparison of overweight, obese, and severely obese adolescents regarding health-related quality of life.

## Discussion

The main findings demonstrated no significant differences for RHR, HRPF, relative fat, lean mass (relative and absolute), and HRQoL among adolescents with overweight, obesity, and severe obesity. However, differences were observed regarding anthropometric variables and blood pressure levels. The results of boys and girls, when analyzed separately, were similar. 

The increased prevalence of high blood pressure associated with an increased degree of excess weight demonstrates the need for special attention to children and adolescents with obesity and severe obesity, considering that high blood pressure has a negative impact on the health of children and adolescents, increasing the risk for cardiovascular disease in adulthood.^2^

There are studies in the literature that have compared metabolic parameters of adolescents classified into different nutritional status. Rizzo *et al* 20 performed a study with 321 adolescents classified according to BMI as overweight, obese, and severely obese and found that risk factors for metabolic syndrome (e.g., HDL-C, insulin resistance, triglycerides, SBP, DBP, and waist circumference) were more frequent in girls with a higher degree of excess weight, when compared to the overweight and obese, except for fasting glucose. For boys, the results were similar for waist circumference, SBP, and DBP; however, there were no significant differences in blood glucose, HDL-C, triglycerides, and insulin resistance. 

Rank *et al* 11 assessed 463 adolescents aged 6 to 19 years and found differences in variables related to cardiovascular risk when they compared moderately obese adolescents with the severely obese. 

For girls, it was found that those with higher BMI also had higher SBP, DBP, insulin resistance, and triglycerides, and lower HDL-C. For males, higher SBP, DBP, LDL-C, insulin resistance, and triglycerides, and lower HDL-C were observed in those classified as severely obese, compared to the moderately obese. In addition to these parameters, the authors also observed that the adipose tissue inflammatory markers (e.g., interleukin-6 and high-sensitivity C-reactive protein) were higher in adolescents with higher degree of excess weight. Adiponectin was also lower in adolescents with severe obesity, when compared with those with a lower degree of obesity. 

In the study by Ricco *et al*,^9^ which compared the presence of risk factors in 84 children and adolescents aged between 6 and 17 years with a diagnosis of overweight and obesity, no differences were found regarding the presence of alterations in the variables total cholesterol, LDL-C, triglycerides, and blood pressure, suggesting the need for attention also to overweight individuals. 

Studies have shown that adolescents with normal weight have higher levels of HRPF when compared to the overweight and obese,^6^^,^ 21 but there appears to be no differences between overweight and obese adolescents.^5^ In this context, the study by Aires *et al* 5 showed that boys and girls with obesity had lower values ​​of strength and cardiorespiratory fitness when compared to normal weight adolescents. In the present study, no differences were found between the groups of adolescents with overweight, obesity, and severe obesity for HRPF variables. 

In practical terms, these results suggest that, regardless of the degree of excess weight, attention to HRPF should not be differentiated, especially regarding cardiorespiratory fitness, which needs to be the focus during treatment due to its association with lower levels of abdominal adiposity,^22^ thus representing a protective factor against cardiovascular risk factors.^6^

Studies have shown that BMI is inversely correlated with the HRQoL of adolescents,^3.16^ and that obese adolescents have lower scores than normal weight individuals for the physical, emotional, social, psychosocial, and total domains.^9^ In the study by Pinhas-Hamiel *et al* 3 conducted with 182 children and adolescents divided according to quartiles of BMI z-score, the physical and social domains of HRQoL were significantly lower, even in children and adolescents with lesser degrees of excess weight, reflecting physical difficulties and social stigmatization that may be already observed in young individuals with the lowest degree of excess weight. 

The present study showed no differences in scores of adolescents with overweight, obesity, and severe obesity, with the scores obtained in the present study similar to those observed in other studies with obese adolescents using the same tool.^16^ Thus, it is possible that the degree of excess weight is not the main determinant factor of HRQoL. In adolescents with excess weight, reduced HRQoL seems to be related to symptoms of depression, anxiety, and low self-esteem, which negatively affect the daily activities of these adolescents.^7.23^

Although the nutritional status classification by BMI divides the adolescents into three different degrees of excess weight, body composition analysis showed a high percentage of fat for all strata of BMI (mean body fat (%)>40%), with no significant differences between them. This suggests that BMI may not be able to identify the differences regarding the excess accumulation of body fat, reinforcing the criticism made by Gallagher *et al*.^24^ Furthermore, BMI, when analyzed alone and individually, may not demonstrate the presence or the impact of comorbidities and functional limitations,^25^^,^ 26 such as metabolic, physical, and psychosocial alterations, which are important to guide decision-making in clinical practice and to provide more complete results on the health of young obese individuals, rather than simply their degree of excess weight.^27^

The results of this study, together with many others in the literature, do not reduce the importance of BMI as a tool for the classification of obesity, especially in epidemiological settings,^28^ as investigations have shown that a five-unit increase in BMI>25 kg/m² is associated with a rise of 29% in mortality from all causes, 41% of vascular mortality, and 210% of diabetes-related mortality.^29^ Moreover, the worsening of metabolic and hemodynamic profile appears to be discriminated by the degree of obesity determined by BMI.^11^^,^ 20 The present analysis only reinforces the idea of using BMI together with other parameters,^27^ as it might not be sensitive enough to identify a lower HRQoL or important cardiorespiratory fitness variables to evaluate the patient with obesity.^30^

This study also has limitations. The analysis was performed with adolescents that sought an intervention program. It is possible that this group has results that differ from those of the general population of adolescents with excess weight; thus the results cannot be extrapolated to the entire pediatric population with excess weight and should be analyzed with caution.

However, while this factor represents a methodological limitation, it is important to understand the physical, clinical, and psychosocial profile of adolescents seeking obesity treatment, serving as basis to determine actions during the intervention process. As further limitation, the level of physical activity of the assessed adolescents was not measured, which might have had some influence on the findings. Another study bias is the lack of a control group with normal weight to compare the results; however, the main objective of this study was to compare different degrees of excess weight aiming to better understand the differences between these groups. In addition, variables that have no specific and objective classification between what would be appropriate and inappropriate for this population (eg: HRQoL) were presented.

In conclusion, the results of this study suggest that adolescents with overweight, obesity, and severe obesity show similar results for the variables RHR, HRPF, relative body fat, lean mass (relative and absolute), and HRQoL.

However, adolescents with a higher degree of excess weight demonstrated higher values ​​of anthropometric variables and blood pressure. These findings provide important practical results and assist in better targeting health interventions regarding the treatment of adolescents, according to the differences and similarities between the different degrees of excess weight. 
