# Supplementary material for: Physical, clinical, and psychosocial parameters of adolescents with different degrees of excess weight
Source: Rev Paul Pediatr. 2014 Dec;32(4):342–50. doi: 10.1016/j.rpped.2014.04.003 (PMC4311788; doi:10.1016/j.rpped.2014.04.003)
Supplement: Translation

# Translation: Parâmetros físicos, clínicos e psicossociais de adolescentes com diferentes graus de excesso de peso☆

**Authors:** Vanessa Drieli Seron Antonini, Danilo Fernandes da Silva, Josiane Aparecida Alves Bianchini, Carlos Andres Lopera, Amanda Caroline Teles Moreira, João Carlos Locateli, Nelson Nardo

**Affiliations:** Universidade Estadual de Maringá (UEM), Maringá, PR, Brasil

**Keywords:** Sobrepeso, Obesidade, Saúde do adolescente, Atividade motora, Qualidade de vida

## Abstract

**OBJETIVO:**

Comparar composição corporal, parâmetros hemodinâmicos, aptidão física relacionada à saúde e qualidade de vida relacionada à saúde em adolescentes com o diagnóstico antropométrico de sobrepeso, obesidade e obesidade grave.

**MÉTODOS:**

Foram selecionados 220 adolescentes com excesso de peso, ingressantes em um programa de intervenção segundo os critérios de inclusão neste programa, baseados na idade, disponibilidade, presença de excesso de peso, local de residência e concordância na participação do estudo. Foram coletadas variáveis antropométricas, hemodinâmicas, aptidão física relacionada à saúde e qualidade de vida relacionada à saúde dos adolescentes. Para a análise de comparação entre os três grupos, foram utilizados testes paramétricos e não paramétricos quando apropriado. A significância foi pré-estabelecido em *p<*0,05.

**RESULTADOS:**

Não houve diferença significativa para a frequência cardíaca de repouso, aptidão física relacionada à saúde, gordura relativa, massa magra (relativa e absoluta) e qualidade de vida relacionada à saúde entre adolescentes com sobrepeso, obesos e obesos graves (*p>*0,05). O peso, índice de massa corporal, circunferência de cintura e de quadril e pressão arterial sistólica aumentaram conforme aumentou o grau de excesso de peso (*p<*0,05). A pressão arterial diastólica do grupo com obesidade grave foi maior que a dos demais grupos (*p<*0,05). Observou-se associação entre o aumento grau de excesso de peso e a prevalência de pressão arterial alterada (sobrepeso: 12,1%; obesidade: 28,1%; obesidade grave: 45,5%; *p<*0,001). Os resultados foram semelhantes quando os sexos foram analisados separadamente.

**CONCLUSÃO:**

Os resultados sugerem que adolescentes com sobrepeso apresentam resultados semelhantes aos obesos e obesos graves nas variáveis analisadas.

## Introdução

Dados recentes do Instituto Brasileiro de Geografia e Estatística (IBGE)^1^ apontam que 20% da população brasileira entre 10 e 19 anos está com excesso de peso (sobrepeso ou obesidade). Essa enfermidade pode trazer complicações para a saúde, como aumento do risco de doenças cardiovasculares ainda na fase escolar,^2^ diabetes tipo 2^3^ e redução do bem-estar físico, emocional e social.^4^

Níveis de aptidão física relacionada à saúde (AFRS) estão inversamente associados com o grau de excesso de peso de crianças e adolescentes.^5^^,^ 6 Aires *et al* 6 observaram correlação inversa entre o Índice de Massa Corporal (IMC) e a máxima capacidade aeróbia em meninos e meninas com sobrepeso e obesidade. Níveis de aptidão cardiorrespiratória e força também são inferiores em adolescentes com excesso de peso, quando comparados a seus pares eutróficos, porém, não há diferença entre indivíduos com sobrepeso em comparação aos obesos.^5^

Outro parâmetro que sofre impacto negativo da obesidade é a qualidade de vida relacionada à saúde (QVRS). Estudos observaram que o excesso de peso tem associação com QVRS mais baixa em adolescentes.^4^^,^ 7 Poeta *et al* 8 verificaram que adolescentes obesos apresentavam piores escores de QVRS para os domínios físicos, social, emocional, psicossocial e total em comparação à adolescentes eutróficos. 

Apesar dos estudos que comparam adolescentes acima do peso com adolescentes com peso normal apresentarem resultados que demonstram a necessidade de atenção especial à população obesa infanto-juvenil, são escassas as comparações entre adolescentes com diferentes graus de excesso de peso. Nesse contexto, Ricco *et al* 9 compararam adolescentes diagnosticados com sobrepeso e aqueles com obesidade e verificaram que os adolescentes com sobrepeso apresentaram riscos à saúde semelhantes aos obesos para os valores de glicemia de jejum, teste de tolerância oral à glicose (OGTT), colesterol total, LDL-colesterol, HDL-colesterol e trigli cérides.

Recentemente, Cole e Lobstein^10^ propuseram pontos de corte baseados no IMC para a classificação de mais um grau de excesso de peso em crianças e adolescentes, sendo este denominado de obesidade grave, baseada no IMC de 35 kg/m² para adultos. Crianças e adolescentes classificadas com obesidade grave apresentam um risco aumentado para síndrome metabólica, resistência à insulina, triglicérides e interleucina-6 comparadas às obesas.^11^ Entretanto, até onde se tem conhecimento, ainda são escassos estudos sobre as diferenças em variáveis antropométricas, composição corporal, parâmetros hemodinâmicos, AFRS e QVRS em adolescentes classificados com sobrepeso, obesidade e obesidade grave, sendo necessário compreender quais são os parâmetros relacionados à saúde que um grau mais elevado de excesso de peso pode influenciar. Conhecer as variáveis que são mais prejudicadas à medida que aumenta o grau de excesso de peso poderá contribuir para estratégias de intervenção na população pediátrica com excesso de peso, sugerindo maior atenção a esses parâmetros, cuja piora se relaciona diretamente ao maior grau de excesso de peso. Por outro lado, determinar as variáveis em que adolescentes com sobrepeso apresentam resultados semelhantes à adolescentes com obesidade e/ou obesidade grave demonstrará a necessidade de se rediscutir políticas de atenção à saúde de adolescentes, que atualmente enfoca o jovem obeso^9^.

Desse modo, o objetivo do estudo foi comparar composição corporal, parâmetros hemodinâmicos, AFRS e QVRS em adolescentes com o diagnóstico antropométrico de sobrepeso, obesidade e obesidade grave, considerando-se todos esses desfechos como principais, dada sua relevância na avaliação do jovem obeso.

## Método

Trata-se de um estudo descritivo transversal. Foram incluídos 220 adolescentes com excesso de peso (sobrepeso, obesidade ou obesidade grave), ingressantes em um Programa Multiprofissional de Tratamento da Obesidade (PMTO) entre os anos de 2009 e 2012. Este é um programa de intervenção que conta com a participação de profissionais de educação física, nutrição, psicologia e pediatria que estão focados em promover mudanças positivas sobre hábitos alimentares e atividade física de adolescentes com excesso de peso, por meio da terapia cognitivo comportamental. Esse programa é oferecido duas vezes ao ano (uma em cada semestre) e tem duração de 16 semanas, sendo seis horas de atividades semanais.

Os critérios de inclusão no programa foram: a) idade variando entre 10 e 18 anos; b) apresentarem disponibilidade para participar das intervenções nos horários e nos dias estipulados; c) apresentarem sobrepeso, obesidade ou obesidade grave, segundo os pontos de corte para o IMC, idade e sexo, propostos por Cole *et al*,^10^ d) residirem em Maringá ou região metropolitana e; e) a concordância e assinatura do Termo de Consentimento Livre e Esclarecido (TCLE) do adolescente e de seu responsável a partir de documento aprovado pelo Comitê de Ética local (parecer nº 463/2009). Os critérios de exclusão foram: a) doenças genéticas ou endócrinas previamente diagnosticadas e informadas ao pediatra; b) consumo em longo prazo de álcool e; c) uso de glicocorticoides e psicotrópicos que poderiam afetar o apetite. Para participação no estudo, foram utilizados os mesmos critérios, exceto o que se refere à disponibilidade para participar das intervenções nos horários e nos dias estipulados. Foram excluídos do estudo, portanto, 59 participantes do programa de intervenção que não preenchiam os critérios acima: um adolescente com diabetes tipo I eutrófico segundo o IMC, um participante comdeficiência intelectual, 40 com idade superior a 18 anos e 17 com idade inferior a 10 anos. Nenhum paciente envolvido no estudo relatava participação prévia em programa regular de exercícios físicos ou de intervenção sistematizado para perda de peso. A única atividade física regular relatada pelos mesmos erai a realizada durante as aulas de educação física escolar.

Foi realizada uma reunião agendada com os interessados em participar do projeto a fim de explicar os objetivos e os tipos de intervenções aos quais os mesmos seriam submetidos. Com os que inicialmente concordaram em participar, foi firmado um TCLE para o programa, contendo as informações das avaliações referentes ao presente estudo. Todas as avaliações do estudo foram conduzidas no período da tarde, entre às 14:00 e 16:00 horas nos espaços utilizados para realização do programa. Todos os adolescentes foram avaliados no *baseline*, antes de iniciarem as atividades do referido programa.

Os adolescentes passaram por uma bateria de avaliações, as quais incluíram a medida do peso corporal, estatura, circunferência de cintura (CC) e circunferência de quadril (CQ). O peso corporal foi medida por meio de uma balança com capacidade para medir até 300 kg e escala de 0,05kg. A estatura foi medida em um estadiômetro com capacidade de medir até 2,30 metros e escala de 0,1cm. O IMC foi calculado a partir da divisão do peso dos adolescentes pela sua altura ao quadrado. As CC e CQ foram medidas com o auxílio de uma fita não extensiva com capacidade de medir até dois metros e escala de 0,1 cm. Foi calculada a relação entre a medida da cintura e do quadril (RCQ).

A avaliação da composição corporal foi feita a partir de um aparelho de bioimpedância elétrica, multifrequencial, octapolar da marca *InBody* modelo 520. Os adolescentes foram aconselhados a seguirem as recomendações descritas por Heyward^12^ para esse tipo de avaliação: jejum de no mínimo 2 horas inclusive de água, urinar cerca de 30 minutos antes da avaliação; abster-se do consumo de bebidas cafeinadas ao longo das últimas 48 horas; evitar esforços físicos vigorosos ao longo das últimas 24 horas; por fim, não utilizar diuréticos ao longo dos últimos sete dias. Foram incluídas na análise as medidas da massa gorda absoluta (MGA) e relativa (MGR) e massa magra absoluta (MMA) e relativa (MMR).

A maturação sexual foi avaliada de acordo com os estágios de Tanner,^13^ por meio do auto-exame. Adolescentes identificados no estágio 1 foram considerados como pré-púberes, nos estágios 2 e 3 como púberes e nos estágios 4 e 5 como pós-púberes.

A medida da frequência cardíaca de repouso (FC_rep_) e da pressão arterial (PA) foi feita após um período de 5 a 10 minutos de repouso, por meio de um esfigmomanômetro eletrônico (Microlife, Argóvia, Suíça) que também fornece a medida da frequência cardíaca. A avaliação foi feita no braço direito, com o tamanho de manguito adequado para o adolescente, sendo a medida feita na posição sentada. A prevalência de valores alterados de pressão arterial foi determinada a partir de critérios específicos para a população em questão.^14^

Os parâmetros da AFRS avaliados foram: flexibilidade, força/resistência da musculatura abdominal, força de preensão manual e a aptidão cardiorrespiratória. 

Para avaliar a flexibilidade, foi aplicado o teste de Sentar-e-alcançar com banco de Wells, o qual os adolescentes deveriam sentar-se, com as pernas estendidas e tentar alcançar a maior distância levando as duas mãos, uma sobre a outra, para frente.^15^ A força/resistência da musculatura abdominal foi avaliada a partir do teste de flexão de tronco, em que os adolescentes deveriam realizar o maior número de repetições do exercício abdominal durante um período de 60 segundos.^15^ A força de preensão manual foi avaliada por um dinamômetro da marca *Takey* modelo *TK 120142* com o adolescente em pé, pernas levemente afastadas lateralmente, braços ao longo do corpo, punho e antebraço pronados e com a escala de medida voltada ao avaliador.^16^ Já a aptidão cardiorrespiratória foi medida a partir do teste de vai-e-vem de 20 metros, teste este iniciado a 8,5km/h e com incrementos progressivos de 0,5 km/h a cada minuto até que o sujeito atingisse exaustão. Os adolescentes foram orientados durante as corridas de 20 metros por um sinal sonoro e por um profissional de Educação Física que participou com o intuito de ajudá-los em relação à familiarização com o procedimento e com o ritmo de corrida. Foram utilizadas nas análises medidas estimadas do consumo máximo de oxigênio (VO_2max_) relativo.^17^

Quanto à qualidade de vida, foi aplicado o questionário genérico PedsQL^TM^ 4.0 para auto-avaliação do adolescente. O questionário apresenta 23 itens que abrangem: 1) dimensão física (oito itens), 2) dimensão emocional (cinco itens), 3) dimensão social (cinco itens), e 4) dimensão escolar (cinco itens). As questões perguntam quanto cada item foi um problema durante o último mês, e os respondentes utilizam uma escala de respostas de cinco níveis (0=nunca é um problema; 1=quase nunca é um problema; 2=algumas vezes é um problema; 3=frequentemente é problema; 4=quase sempre é um problema). Os itens foram pontuados inversamente e transpostos linearmente para uma escala de 0-100 (0=100, 1=75, 2=50, 3=25, 4=0); assim, quanto maior o escore, melhor a QVRS. Os escores da escala foram obtidos conforme padronização dos proponentes. Para criar um escore sumário dos escores de saúde psicossocial (15 itens), a média foi computada como a soma dos itens respondidos nas escalas das dimensões emocional, social e escolar dividida pelo número de itens. Para criar um escore sumário da qualidade de vida total, os 23 itens foram computados, o que abrange os quatro domínios do instrumento.^18^ Este questionário foi validado para língua portuguesa por Klatchoian *et al* para crianças e adolescentes (idade entre 2 e 18 anos).^19^ Sua aplicação aconteceu em uma sala de aula com capacidade para cerca de 30 adolescentes, com o auxílio de pelo menos dois avaliadores.

O cálculo do tamanho da amostra foi feito com base em um poder de 80%, um alfa de 5% e uma diferença entre o IMC de adolescentes com sobrepeso (25,10±2,62 kg/m²) e adolescentes com obesidade grave (33,99±5,17 kg/m²), de acordo com os resultados observados por Rizzo *et al*.^20^ A partir desse cálculo, o tamanho da amostra para cada grupo deveria ser de pelo menos 31 adolescentes. Foi utilizado o teste de Shapiro-Wilk para a verificação da normalidade e teste de Levene para verificação da homogeneidade. Os dados foram apresentados em média e desvio padrão, sendo que para a análise de comparação entre os três grupos, de acordo com o estado nutricional, foram utilizados o teste de Kruskal-Wallis para dados que não apresentaram normalidade e/ou homegeneidade e a ANOVA *one-way* para dados que apresentaram normalidade e/ou homogeneidade. Havendo diferenças no teste de Kruskal-Wallis, foi aplicado o teste de comparações múltiplas LSD, enquanto que para as diferenças observadas pela ANOVA *one-way* foi aplicado o teste de comparações múltiplas de Bonferroni. As análises foram feitas com auxílio do *software* estatístico SPSS versão 13.0. O nível de significância foi pré-estabelecido em *p<*0,05.

## Resultados

Dos 220 adolescentes avaliados, 58 (26,4%) foram classificados pelo IMC com sobrepeso, 96 (43,6%) com obesidade e 66 (30,0%) com obesidade grave. No grupo de adolescentes com sobrepeso, 34 (58,6%) adolescentes eram meninas, enquanto que nos grupos de obesos e obesos graves, 50 (52,1%) e 32 (48,5%) adolescentes eram do sexo feminino. A idade dos adolescentes com sobrepeso, obesidade e obesidade grave era de 13,2±1,9 anos, 13,1±1,9 anos e 13,3±1,8 anos, sem diferenças significantes (*p=*0,646). A maturação sexual foi avaliada em uma amostra parcial (n=127). No grupo de adolescentes com sobrepeso, 57,5% eram pós-púberes e 42,5% eram púberes. No grupo de adolescentes obesos, 50% eram pós-púberes e 50% púberes. Quanto aos adolescentes obesos graves, 60% eram pós-púberes, 37,1% eram púberes e 2,9% eram pré-púberes. Não foi observada associação entre o estágio de maturação sexual e o grau de excesso de peso dos adolescentes (*p=*0,949). 

Na amostra total, foram encontradas diferenças estatísticas entre os grupos para as variáveis, peso corporal, IMC, CC, CQ e PAS, em que os adolescentes com sobrepeso apresentaram valores menores que o grupo de obesos e obesos graves. Para as variáveis RCQ e PAD não foram observadas diferenças entre o grupo de sobrepeso e obesidade, porém apenas entre os adolescentes com sobrepeso e os obesos graves. Nenhuma diferença foi observada para a estatura e FCrep. Os resultados para os sexos masculino e feminino analisados separadamente foram semelhantes (tabela 1). Dada a diferença observada para a PAS e PAD entre os graus de excesso de peso, foi determinada a prevalência de pressão arterial elevada na amostra total e de acordo com o sexo do adolescente. Notou-se que 12,1%, 12,5% e 11,8% dos adolescentes da amostra total, meninos e meninas com sobrepeso também apresentavam a pressão arterial alterada. Para os adolescentes obesos, a prevalência de alteração foi de 28,1%, 39,1% e 18,0% em toda a amostra, nos meninos e nas meninas, respectivamente. Para os adolescentes com obesidade grave, a prevalência de alteração foi de 45,5%, 47,1% e 43,8% na amostra total, meninos e meninas. O grau de excesso de peso se associou à prevalência de pressão arterial elevada nas três condições analisadas (amostra total: *p<*0,001; meninos: *p=*0,009; meninas: *p=*0,002).

**Tabela 1:** Comparação entre adolescentes com sobrepeso, obesos e obesos graves, para as variáveis antropométricas e parâmetros hemodinâmicos

A tabela 2 apresenta os valores encontrados para as variáveis de composição corporal e AFRS e, na tabela 3, estão os resultados encontrados para aos domínios da QVRS. Não houve diferença significante entre os três grupos comparados, exceto para a gordura corporal (kg), em que o grupo com sobrepeso apresentou resultados significativamente menores que os adolescentes classificados com obesidade grave. Quando separados pelo sexo, nenhuma diferença para nenhum dos parâmetros das tabelas 2 e 3 foi observada.

**Tabela 2:** Comparação entre adolescentes com sobrepeso, obesos e obesos graves, para as variáveis de aptidão física relacionada à saúde e composição corporal

**Tabela 3:** Comparação entre adolescentes com sobrepeso, obesos e obesos graves, em relação à Qualidade de vida Relacionada à Saúde

## Discussão

Os principais achados demonstram não haver diferença significativa para a FCrep, AFRS, gordura relativa, massa magra (relativa e absoluta) e QVRS entre adolescentes com sobrepeso, obesos e obesos graves. Entretanto, foram encontradas diferenças para as variáveis antropométricas e pressão arterial. Os resultados de meninos e meninas analisados separadamente foram semelhantes.

O aumento da prevalência de pressão arterial elevada associado ao aumento do grau de excesso de peso demonstra a necessidade de atenção especial à população infanto-juvenil com obesidade e obesidade grave, tendo em vista que a pressão arterial elevada tem um impacto negativo sobre a saúde de crianças e adolescentes, aumentando o risco para doenças cardiovasculares na fase adulta.^2^

Na literatura, há trabalhos que compararam parâmetros metabólicos de adolescentes classificados em diferentes estados nutricionais. Rizzo *et al* 20 realizaram um estudo com 321 adolescentes categorizados a partir do IMC, como adolescentes com sobrepeso, obesos e obesos graves e observaram que fatores de risco para síndrome metabólica (ex.: HDL-c, resistência à insulina, triglicérides, PAS, PAD e circunferência de cintura) foram mais frequentes nas meninas com grau mais elevado de excesso de peso, comparadas às portadoras de sobrepesoe obesas, com exceção da glicemia de jejum. No caso dos meninos, os resultados foram similares para a circunferência de cintura, PAS e PAD, entretanto, não houve diferenças significativas para glicemia, HDL-c, resistência à insulina e triglicérides.

Rank *et al* 11 avaliaram 463 adolescentes entre seis e 19 anos e encontraram diferenças nas variáveis relacionadas a risco cardiovascular quando comparados adolescentes moderadamente obesos com obesos graves. Para as meninas, verificou-se que aquelas com valores mais elevados de IMC também apresentaram maior PAS, PAD, resistência à insulina e triglicerídeos e menor HDL-c. Para o sexo masculino, foram observados valores maiores de PAS, PAD, LDL-c, resistência à insulina e triglicérides e menores de HDL-c para aqueles classificados como obesos graves, em comparação aos moderados. Somado a esses parâmetros, os autores também observaram que marcadores inflamatórios do tecido adiposo (ex.: interleucina-6 e proteína C-reativa de alta sensibilidade) estão mais elevados nos adolescentes com maior grau de excesso de peso. A adiponectina também foi menor nos adolescentes com obesidade grave, comparados àqueles com grau menos elevado de obesidade.

No estudo de Ricco *et al*,^9^ em que foi comparada a presença de fatores de riscos em 84 crianças e adolescentes entre seis e 17 anos com diagnóstico de sobrepeso e obesidade, não foram encontradas diferenças para a presença de alterações nas variáveis, colesterol total, LDL-c, triglicerídeos e pressão arterial, sugerindo assim a necessidade de atenção também aos indivíduos com sobrepeso.

Estudos demonstraram que adolescentes eutróficos apresentam melhores níveis de AFRS em comparação aos adolescentes com sobrepeso e obesos,^6^^,^ 21 porém parece não haver diferenças entre adolescentes com sobrepeso e obesos.^5^ Neste contexto, o estudo conduzido por Aires *et al* 5 demonstrou que meninos e meninas com obesidade apresentaram menores valores de força e aptidão cardiorrespiratória quando comparados a adolescentes com peso normal. No presente estudo, não foram encontradas diferenças entre os grupos de adolescentes com sobrepeso, obesos e obesos graves para as variáveis de AFRS. Em termos práticos, esses resultados sugerem que, independentemente do grau de excesso de peso, a atenção à AFRS não deve ser diferenciada, em especial para a aptidão cardiorrespiratória, que precisa ser enfocada durante o tratamento devido à sua associação com níveis baixos de adiposidade abdominal,^22^ representando assim um fator protetor contra fatores de risco cardiovascular.^6^

Estudos demonstram que o IMC é inversamente correlacionado à QVRS de adolescentes,^3^^,^ 16 sendo que adolescentes obesos apresentam escores mais baixos que adolescentes eutróficos para os domínios físico, emocional, social, psicossocial e total.^9^ No estudo de Pinhas-Hamiel *et al*,^3^ realizado com 182 crianças e adolescentes divididos de acordo com os quartis do escore Z do IMC, os domínios físico e social da QVRS foram significativamente menores mesmo nas crianças e adolescentes com menor grau de excesso de peso, refletindo dificuldades físicas e estigmatização social que podem já estar presentes em jovens com grau mais leve de excesso de peso. No presente estudo não foram encontradas diferenças nos escores de adolescentes com sobrepeso, obesos e obesos graves, sendo a pontuação obtida no presente estudo similar à observada em outras análises feitas com adolescentes obesos a partir do mesmo instrumento.^16^ Assim, é possível que o grau de excesso de peso não seja o principal fator determinante da QVRS. Em adolescentes com excesso de peso, a redução da QVRS parece ter relação com sintomas de depressão, ansiedade, baixa autoestima, influenciando negativamente as atividades diárias desses adolescentes.^7^^,^ 23

Embora a classificação do estado nutricional pelo IMC separe os adolescentes em três diferentes graus de excesso de peso, a análise da composição corporal revelou percentual de gordura elevado para todos os estratos do IMC (Gordura (%) média > 40%), sem diferenças significativas entre eles. Isso sugere que o IMC pode não ser capaz de identificar as diferenças quanto ao acúmulo em excesso de gordura corporal, reforçando a crítica feita por Gallagher *et al.* 24 Ademais, o IMC, quando analisado isolada e individualmente, pode não demonstrar a presença ou o impacto de comorbidades ou limitações funcionais,^25^^,^ 26 como alterações metabólicas, físicas e psicossociais, importantes para guiar decisões na prática clínica e fornecerem resultados mais completos sobre a saúde do jovem obeso do que apenas seu grau de excesso de peso.^27^

Os resultados deste estudo, em somatório com outros mais presentes na literatura, não reduzem a importância do IMC como uma ferramenta para classificação da obesidade, especialmente em cenários epidemiológicos,^28^ até mesmo porque pesquisas já demonstraram que aumento de cinco unidades no IMC acima de 25 kg/m² está associado à elevação de 29% da mortalidade por todas as causas, 41% da mortalidade vascular e 210% da mortalidade relacionada ao diabetes.^29^ Também a piora no perfil metabólico e hemodinâmico parece ser discriminada pelo grau de obesidade determinado pelo IMC.^11^^,^ 20 A presente análise apenas reforça a ideia de se utilizar o IMC em conjunto com outros parâmetros,^27^ pois o mesmo pode não ser sensível o suficiente para identificar uma baixa QVRS ou aptidão cardiorrespiratória, variáveis importantes para a avaliar o paciente com obesidade.^30^

O presente estudo também apresenta limitações. A análise foi realizada com adolescentes que procuraram um programa de intervenção. É possível que esse grupo apresente resultados que diferem da população geral de adolescentes com excesso de peso; deste modo os resultados não podem ser extrapolados para toda população pediátrica com excesso de peso e devem ser analisados com cautela. No entanto, ao mesmo tempo que esse fator representa uma limitação metodológica, ele é relevante para compreender o perfil físico, clínico e psicossocial de adolescentes que buscam auxílio para o tratamento da obesidade, servindo de base para determinar ações durante o processo de intervenção. Como limitação adicional, não foi mensurado o nível de atividade física dos adolescentes avaliados, que poderia ter algum tipo de influência sobre os achados. Outro viés do estudo é a não utilização de um grupo controle eutrófico para comparar os resultados; porém, o objetivo principal do presente estudo foi comparar diferentes graus de excesso de peso para melhor compreensão das diferenças entre esses grupos. Ademais, foram apresentadas variáveis que não têm classificação específica e objetiva entre o que seria adequado e inadequado para essa população (ex.: QVRS).

Em conclusão, os resultados do presente estudo sugerem que adolescentes com sobrepeso, obesos e obesos graves apresentam resultados semelhantes para as variáveis FCrep, AFRS, gordura relativa, massa magra (relativa e absoluta) e QVRS. No entanto, os adolescentes com grau mais elevado de excesso de peso apresentaram maiores valores para as variáveis antropométricas e pressão arterial. Estes achados fornecem resultados práticos importantes e auxiliam no melhor direcionamento das ações em saúde referentes ao tratamento de adolescentes, de acordo com as diferenças e semelhanças entre os diferentes graus de excesso de peso.
